# Improving Socially Supported Serious Illness Care for LGBTQIA+ People: A Feasiblity Study

**DOI:** 10.1093/geroni/igaf122.2403

**Published:** 2025-12-31

**Authors:** Raeann LeBlanc, Susan Shaw

**Affiliations:** University of Massachusetts Amherst, Amherst, Massachusetts, United States; University of Massachusetts Amherst, Amherst, Massachusetts, United States

## Abstract

End-of-life doula care is non-medical care centered on positive regard and pragmatic emotional support to address social isolation, a complex issue in living with serious illness in the LGBTQIA+ context. The “Doula Pride” program centers social support for LGBTQIA+ older people with serious illness using a community engaged supportive network approach. Through training and social connection, Doula Pride aimed to decrease social isolation and bolster community based social capital. This pilot intervention used a multi-methods design to test feasibility among a cohort of LGBTQIA+ doula informed carers who completed a 4-week training matched with LGBTQIA+ persons with serious illness from a rural community. Surveys and interviews evaluated self-efficacy, social support, social isolation, social networks, and social capital at the individual and community levels. Thirteen female LGBTQIA+ doula-pride informed carers completed training. Just over half matched with an LGBTQ+ older adult in the community living with serious illness. A total of twenty-six visits were completed during the 4-month practice period. The processes of community engaged planning, curriculum design, recruiting and matching carers, and lessons learned in the process, will be discussed. The “Doula Pride” community engaged program fostered social support among LGBTQIA+ people with serious illness and their carers using an innovative local network approach to address holistic social support and decreased social isolation. Our results suggest it is feasible to sustain this community program through close LGBTQIA+ community engagement.

